# Individual Responses to an 8-Week Neuromuscular Training Intervention in Trained Pre-Pubescent Female Artistic Gymnasts

**DOI:** 10.3390/sports6040128

**Published:** 2018-10-24

**Authors:** Sylvia Moeskops, Paul J. Read, Jon L. Oliver, Rhodri S. Lloyd

**Affiliations:** 1 Youth Physical Development Centre, Cardiff School of Sport & Health Sciences, Cardiff Metropolitan University, Cardiff CF23 6XD, UK; Paul.Read@aspetar.com (P.J.R.); joliver@cardiffmet.ac.uk (J.L.O.); rlloyd@cardiffmet.ac.uk (R.S.L.); 2 Athlete Health and Performance Research Centre, Aspetar Orthopaedic and Sports Medicine Hospital, Doha 29222, Qatar; 3 Sport Performance Research Institute New Zealand (SPRINZ), AUT University, Rosedale, Auckland 1142, New Zealand; 4 Centre for Sport Science and Human Performance, Waikato Institute of Technology, Hamilton 3204, New Zealand

**Keywords:** leg stiffness, reactive strength index, youth, gymnastics, movement competency

## Abstract

This study examined individual responses in leg stiffness, reactive strength index (RSI), movement proficiency (deep overhead squat and in-line lunge), and trunk muscular endurance (flexor and extensor tests) in young female gymnasts following an 8-week neuromuscular training intervention. Thirty-four pre-peak height velocity (PHV) female gymnasts were divided into either an experimental group (EXP *n* = 17) or control group (CON *n* = 17). The EXP replaced their normal gymnastics physical preparation with a neuromuscular training program, while the CON continued with their habitual gymnastics program. Chi square analysis showed that the EXP resulted in significantly more positive responders compared to CON for measures of leg stiffness (41% versus 12% responded positively), extensor muscular endurance, (76% versus 29%), and competency in the deep overhead squat, (76% versus 29%) and in-line lunge (left lead leg) (65% versus 18%). Conversely, the number of positive responders for RSI (53% versus 61%), the flexor endurance test (88% versus 53%), and the right in-line lunge (47% versus 35%) were not significantly different between groups. These findings suggest that most young gymnasts responded positively to neuromuscular training from the perspective of improving movement proficiency and trunk endurance; however, changes in leg stiffness and RSI were more variable and may require higher intensities to realise further adaptations.

## 1. Introduction

Female artistic gymnastics is an early specialisation sport, typically involving high volumes and intensities of training during the pre-pubertal years aimed at mastering the performance of complex skills [1]. Since relative strength is a more important determinant of gymnastics performance than absolute strength [2], it is unsurprising that many coaches traditionally use body-weight training in the form of circuits and repetition of skills to physically prepare gymnasts [3]. However, while this training modality is often effective in developing highly sport-specific qualities, the addition of developmentally appropriate neuromuscular training could offer pre-pubertal gymnasts’ numerous benefits, that surpass body-weight and skills training alone [4,5,6]. Research suggests that neuromuscular training which integrates the development of fundamental movement skills with muscular strength and power could facilitate technical competency of sport-specific skills [7,8], assist in correcting aberrant movement patterns [9,10,11], and help promote long-term participation in competitive and recreational sport [8].

Gymnastics involves a series of rebounding activities which utilise various expressions of stretch-shortening cycle (SSC) activity, ranging from slow-SSC (ground contact time >250 ms e.g., acrobatic skills on the beam) to fast-SSC activity (ground contact time <250 ms e.g., tumbling) [12]. The sport also requires gymnasts to isometrically hold shapes during the performance of individual static skills (e.g., a planche) and during a series of dynamic skills (e.g., giants on the bars) [13]. When measuring different expressions of SSC function, selecting a test protocol that represents the specific type of SSC action is recommended, owing to the different mechanisms involved in fast- and slow-SSC activity [14,15,16]. Current methods of examining neuromuscular training interventions on fast-SSC function in youth have included reactive strength index (RSI) and leg stiffness, from drop jumps [17] and hopping tasks [18,19,20].

Recent research suggests that supplementary neuromuscular training (e.g., inclusive of resistance training and plyometric training) targeting the development of muscular strength could improve gymnasts’ ability to utilize their SSC [5], when performing explosive skills such as vaulting or tumbling [5,21]. Due to the potential confounding effect of skill proficiency when using actual sporting techniques, measuring RSI and leg stiffness in lab-based conditions can provide an insight into the underlying mechanisms of the gymnasts’ SSC abilities and thus, inform coaches training programmes. However, very few studies have investigated the effects of neuromuscular training on measures of RSI and leg stiffness in young female gymnasts [22]. Furthermore, the importance of youth developing a high level of movement proficiency across a range of basic motor skills has been well documented [8], as correct technique may enable a more effective transfer of force when performing more dynamic, complex skills [23]. Specific tests from the functional movement screen (FMS) (in-line lunge and deep overhead squat) [24,25] have been used to assess movement pattern proficiency, and have been correlated with measures of physical performance such as RSI and squat jump height in young athletes [23]. Since most gymnastics skills are underpinned by basic athletic motor skill competencies (AMSCs) (e.g., jumping, landing, trunk bracing), assessing the efficacy of neuromuscular training on young female gymnasts’ movement proficiency would appear noteworthy.

While research has demonstrated that youth can respond positively to forms of neuromuscular training [7,11,20,26], these studies have typically considered the group response to training interventions. However, research that solely examines training responses at a group level will fail to account for the differentiation between positive responders, non-responders, and negative responders. Given that individuals of the same chronological age can differ markedly with respect to growth and maturation, training experience and cognitive development [27], the responsiveness to training has the potential to differ greatly within a cohort of young athletes. Research has indicated that adaptations to sprinting and jumping performance in response to resistance-based interventions are influenced by maturation in young males [19,20]. Thus, large variations in responsiveness to training in youth are probable, evidencing the need to examine how young athletes respond to training at an individual level in other sports.

Unfortunately, very few studies in youth have examined the individual responses to neuromuscular-based training programs. One recent study found that in school-aged boys, individual responsiveness to training was influenced by both the mode of resistance training and maturity status [19]. However, the effects of similar training interventions in young female gymnasts remains unclear. These athletes are routinely exposed to a high amount of plyometric and body weight strength type activities; thus, examining the individual responsiveness to neuromuscular training could provide a useful insight into the trainability of this unique population. The aim of this study was to determine the individual responsiveness of pre-pubertal female gymnasts participating in an 8-week neuromuscular training programme, on measures of SSC, trunk muscular endurance, and movement quality.

## 2. Materials and Methods

### 2.1. Subjects

Thirty-four female artistic gymnasts aged between 6 and 12 years (*n* = 17 experimental group and *n* = 17 control group) volunteered to take part in this study. All gymnasts were from the same gymnastics club and were assigned to either the experimental (EXP) or control (CON) group based on the days they attended gymnastics training (i.e., those training on a Monday and Wednesday = EXP and those training Tuesday and Thursday = CON). Participant characteristics for both EXP and CON groups are presented in Table 1. Each participant had a minimum of 1 year’s artistic gymnastic experience, trained between 6 and 20 h per week, and reported no injuries at the time of testing. All gymnasts were of a similar standard and classified by the gymnastics coaches as being at an intermediate standard. This was based on the coach’s evaluation of gymnastics competency and the level of competition the gymnasts had competed at. Both groups of gymnasts were in a competitive phase of training, which involved 2–3 competitions over a 4–6-week period, and none of the participants had previously engaged in formalized strength and conditioning programs. The study was approved by Cardiff Metropolitan University’s Research Ethics Committee (approval code 13/7/01U), and informed parental consent and participant assent were obtained in advance of the study commencing.

### 2.2. Procedures

#### 2.2.1. Familiarization Session

Anthropometric data were collected including standing and seated height using a stadiometer to the nearest 0.1 cm (SECA, 321, Vogel & Halke, Hamburg, Germany), and body mass using scales to the nearest 0.1 kg (SECA, 321, Vogel & Halke, Hamburg, Germany). Standing height, seated height, and body mass were entered into a validated sex-specific prediction equation [28] to determine participants’ maturity status as years from peak height velocity (PHV), which refers to the maximum rate of growth during the adolescent growth spurt [29]. Functional leg dominance was determined by the most frequently used leg during the step up and balance recovery tests [30]. Participants were then provided with the opportunity to familiarize themselves with all testing protocols until the lead researcher was satisfied with the gymnast’s technical competency.

#### 2.2.2. Testing Procedures

The EXP group followed an 8-week neuromuscular training program, consisting of two weekly sessions that lasted approximately 35 min each, which replaced their normal gymnastics conditioning. The CON group continued with their usual gymnastics program and did not receive any formalized strength and conditioning provision. The gymnastics programme for the CON group involved 35 min of gymnastics-specific conditioning (e.g., shaping and gymnastics skill-based exercises), which was delivered by a club gymnastics coach and not the principle researcher. All testing sessions took place at the local gymnastics club where the participants trained. Both EXP and CON groups completed the same battery of tests at the beginning and end of the 8-week program (pre- and post-testing occurred 1 week either side of the program), and the same researcher administered each protocol. Gymnasts performed all tests barefoot wearing a gymnastics leotard. After a standardised 10-min dynamic warm up and practise trials, gymnasts performed the test battery in the following order: deep squat and in-line lunge (left and right lead leg), sub-maximal hopping, drop jump, and trunk endurance holds. All testing protocols have previously been shown to be reliable within the paedatric literature [21,31,32]. The gymnasts performed three trials of the deep squat, in-line lunge and drop jump protocols, while a single trial of the endurance hold tests [33] and the sub-maximal hopping protocol [31] were performed according to previously reported guidelines. Participants were afforded rest periods of 60 s between trials and 5 min between each protocol to limit the effects of fatigue on the performance variables.

#### 2.2.3. Movement Proficiency

Participants were screened using a modified version of the FMS [25], whereby two of the protocols were chosen to assess movement proficiency; the deep overhead squat and the in-line lunge. These specific tests were selected from the full FMS due to their significant correlations with measures of physical performance in youth [23]. The tests also provided an assessment of the gymnasts’ bilateral and unilateral lower limb movements, both of which are integral to gymnastics performance [6]. Each gymnast performed three trials of both tests and were scored real-time using the 4-point scale from the FMS movement criteria (the rater had 2 years of experience using this screening tool) [24,25]. Testing guidelines for scoring were followed, whereby the highest score of the three trials was recorded for further analysis [25]. For the in-line lunge test, scores were recorded for the gymnasts’ left and right leg, which referred to the leading leg during the protocol.

#### 2.2.4. Sub-Maximal Hopping

Leg stiffness (kN·m^−1^) was determined via a sub-maximal hopping test collected on a mobile contact mat (Smart-jump, Fusion Sport, Brisbane, Australia). The Equation (A1) for leg stiffness can be found in Appendix A [18,31]. Each participant performed one trial of 20 consecutive hops at a frequency of 2.5 Hz, which was maintained via a quartz metronome [31]. The gymnasts were instructed to keep their hands on their hips and rebound with their legs extended in time to the metronome. All contact mat data were collected via a PDA (iPAQ, Hewlett Packard, Palo Alto, CA, USA) and later exported as an Excel file for analysis. Ten consecutive “acceptable hops” were then selected for analysis, whereby the participants’ hopping frequency was closest to the designated metronome rate [31]. Variables obtained from contact mat data enabled the calculation of vertical leg stiffness using equations and methods previously reported in youth-based studies [31].

#### 2.2.5. Drop Jumps

All drop jumps were performed on a portable force plate (AMTI, Accupower, Boston, MA, USA) from a box height of 20 centimetres [34]. Participants were instructed to step off the box and rebound as high and as quickly as possible. The gymnasts were permitted to use an arm swing [35] to mimic the activities they perform in their gymnastics practise, and were instructed to keep their legs extended during the flight phase of the jump. Trials where the gymnasts noticeably stepped down or jumped up from the box were discounted and repeated. Using previously established methods [35,36], the raw vertical force-time data were used to calculate the variables needed to determine reactive strength index (RSI). The Equation (A2) [35,36] for calculating RSI can be found in Appendix A.

#### 2.2.6. Trunk Muscular Endurance Hold Tests

To assess trunk muscular endurance, each gymnast performed the Biering-Sorenson test to assess trunk extensor and flexor endurance [37], isometrically holding test positions until technical failure. Both tests have previously been used in gymnastics-based literature [33]. The extensor muscular endurance test required the participants to lie in a prone position, with their anterior-superior iliac spine aligned to the edge of the testing box, while a coach held their lower extremities [33,37]. Participants were then asked to cross their arms and place their hands on their shoulders, before lifting their torso to the horizontal testing position. For the flexor endurance test, participants assumed a seated position with 90° of flexion at the hips and knees [33]. The same arm position as the extensor test was adopted by the gymnasts and a 60° back angle was measured for the testing position using a handheld goniometer (plastic 12 inch, 66 fit). The principal investigator observed both tests from a lateral view to check the gymnasts’ technique was being maintained. The gymnasts performed a single trial of each isometric test, where maximum time held was recorded using a stop watch to the nearest 0.1 of a second. To reduce testing bias, participants were not informed of their results following baseline testing or prior to post testing [33].

### 2.3. Training Program

To ensure technical execution of exercises was maintained throughout, the program was carefully supervised by the lead researcher who was a certified strength and conditioning specialist and qualified gymnastics coach. Program design adopted an integrative approach to neuromuscular training based on previous training interventions shown to be effective with inexperienced youth [7,10,18,38]. Specifically, the program included elements of trunk muscular endurance, movement competency, dynamic stabilization, plyometrics, and strength training. The content of the sessions was divided into three sections: trunk muscular endurance (approximately 5-min), movement preparation (approximately 15-min) and basic resistance training exercises (approximately 15-min), a more detailed overview can be found in Table 2. The intensity was increased for participants that were technically competent using resistance in the form of: body weight, resistance bands, medicine balls and free weights. Specifically, individuals were progressed on a session-by-session basis using small increments (increments < 5 kg) in load, depending on the gymnasts’ technical competency. The trunk conditioning exercises were largely isometric and based around the shapes required for gymnastics, such as dish and arch shapes and bracing activities for handstands. In the movement preparation part of the program, fundamental movement skills were developed alongside force absorption/control landing activities. The resistance training element concentrated on addressing the gymnasts’ lower body force expression and technical competency through both unilateral and bilateral exercises. Emphasis was placed on correct lower-limb biomechanics during all exercises to facilitate the safe execution of skills.

### 2.4. Statistical Analyses

Descriptive statistics (means ± standard deviations) were calculated for all pre- and post-data for each group. A multivariate analysis of covariance (MANCOVA) was used to control for baseline differences in group anthropometrics (multivariate comprised of body mass, height and leg length) for each performance variable. The assumption of normality was assessed via the Shapiro-Wilk test. A 2 × 2 repeated-measure analysis of variance (ANOVA) (group × time) was then used to examine the group changes in performance for each variable. Sphericity was assessed using Mauchly’s Test and where violated, a Greenhouse-Geisser adjustment was implemented. Percentage change from baseline testing was calculated for all individuals in each of the performance variables. The group percentage changes in performance were examined for each variable using an independent *t*-test.

In order to examine the individual responsiveness to the training intervention, the smallest worthwhile change (SWC) was calculated as 0.2 of the between subject SD for the total sample, using pre-intervention data. The SWC was expressed as a percentage of the group mean and a frequency count was then used to establish the number of individuals who made changes greater than the SWC, identifying those individuals who responded positively in performance. Chi-squared analysis was used to evaluate between-group differences for the number of positive responders for each performance variable. Analysis of the standardized residuals was used to identify frequencies that would be considered larger in magnitude than might be expected by chance [39], and was identified as significantly different using the >1.96 criteria [40]. Descriptive statistics, repeated-measures ANOVAs and chi-squared analysis were all computed using SPSS Statistics v.22, with statistical significance set at an alpha level of *p* < 0.05. Median values and percentages of individual scores for the deep overhead squat and in-line lunge data were calculated via Microsoft Excel for Mac version 15.35.

## 3. Results

The results from the MANCOVA did not reveal any significant between-group differences for any performance variable at baseline testing when maturity was controlled for as a multivariate factor (*p* < 0.05). The between-group results from the repeated-measures ANOVA and percentage change are displayed in Table 3. The EXP group showed significant improvements in the flexor and extensor muscular endurance, when compared to the CON group (*p* < 0.05). Neither group showed significant improvements in RSI; however, the control group showed a significant reduction in leg stiffness.

**Figure 1:**
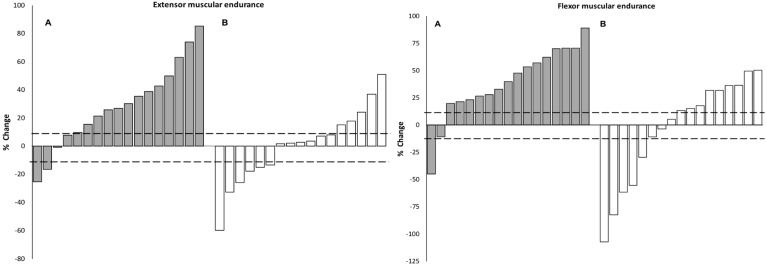
Individual percentage change in extensor and flexor muscular endurance for the experimental (grey bars) and control (open bars) subjects. The horizontal line represents the smallest worthwhile change for all training groups combined.

**Figure 2:**
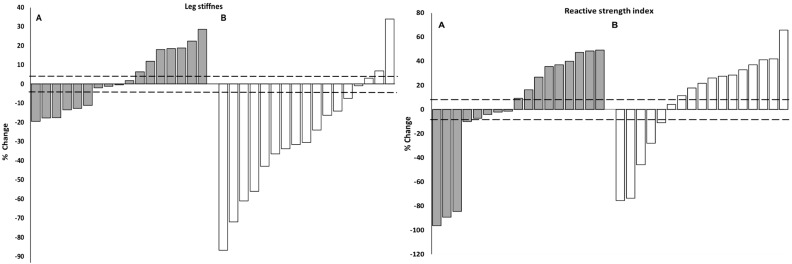
Individual percentage change in leg stiffness and RSI for the experimental (grey bars) and control (open bars) subjects. The horizontal line represents the smallest worthwhile change for all training groups combined.

The individual percentage change and the number of individuals that responded positively (*n* > SWC) for the flexor and extensor endurance test, leg stiffness, and RSI is shown in Table 3, and Figure 1 and Figure 2. Chi-squared analysis revealed there were significantly more positive responders than would be expected by chance for leg stiffness and extensor muscular endurance in the EXP group, compared to the CON group (standardized residual values *n* > 1.96). Specifically, for leg stiffness, the number of positive responders and non-responders were greater in the EXP group (41% and 24%) versus the CON group (12% and 12%); while the EXP also showed a lower number of negative responders (35% versus 76%). A similar pattern of positive responders, non-responders, and negative responders emerged for extensor muscular endurance (76%, 12%, 12% versus 29%, 35%, 35%, respectively).

Over 50% of individuals made improvements greater than the SWC in the flexor muscular endurance test and RSI in both groups; however, analysis of the standardized residuals revealed that neither group’s number of positive responders were significantly greater than what would be expected by chance. Of note, the distribution of positive responders, non-responders and negative responders for the flexor muscular endurance would suggest overall, the EXP group had a more favourable response (88%, 6%, 6%) compared to the CON (53%, 18%, 29%). Conversely, the opposite pattern was evident for RSI (EXP = 53%, 23.5%, 23.5% versus CON = 65%, 6%, 29%, respectively).

The median values and the distribution of test scores for the deep squat and in-line lunge are presented in Table 4 for both groups. The EXP group demonstrated improvements in median values, improving from a score of 1 to 2 in both the deep squat, left and right in-line lunge. The distribution of the CON group’s individual scores for the deep overhead squat and in-line lunge also improved; however, the median values of the pooled data remained the same, with a score of 2 for each test. Chi-squared analysis revealed there were significantly more positive responders than expected in the EXP versus CON group for the deep overhead squat (72% versus 29%) and the in-line lunge (left) (76% versus 18%). However, for the in-line lunge (right) test, neither group’s number of positive responders were significantly greater than the expected frequency count (EXP group = 47% versus CON group = 35%).

## 4. Discussion

The aim of this study was to examine individual responses in leg stiffness, reactive strength index (RSI), movement proficiency (deep overhead squat and in-line lunge), and trunk muscular endurance (flexor and extensor tests) in young female gymnasts following an 8-week neuromuscular training intervention. The main finding of this study was that an 8-week neuromuscular training program resulted in a higher percentage of responders in in pre-pubertal female gymnasts in most performance variables. The EXP group had a higher number of positive responders in both trunk endurance tests, the deep overhead squat and the in-line lunge tests, and had a significantly greater number of positive responders in leg stiffness compared with the CON. The number of positive responders for RSI were not significantly greater in either group. These findings suggest that supplementary neuromuscular training can provide additional benefits to pre-pubertal female gymnasts’ training programs. However, given the range of positive responders, non-responders and negative responders across all performance variables, alternative training prescription (e.g., different exercise selections, higher training intensities) may be required to promote more homogenous improvements in reactive strength and stiffness qualities.

The group results from the deep squat and in-line lunge tests suggest that the training program was effective in eliciting improvements in the EXP group, with median values improving from a score of 1 to 2 in the deep squat and in-line lunge (consistent on both left and right limbs). While the distribution of the CON group’s individual scores showed improvement, the median values of the pooled data remained the same across all three tests. Participation in gymnastics can develop a variety of motor skills [41], but this study highlights the potential benefits of exposing young trained gymnasts to movement skill training that is different to their habitual gymnastics training. Furthermore, as the neuromuscular training program included a variety of squatting and lunging movement patterns, it is likely that the improvements in FMS scores of the EXP group were the result of a specific response to the imposed training demand.

In terms of the extensor and flexor muscular endurance tests, the EXP group made significant improvements in both tests, when compared with the CON. Previous research has shown participation in gymnastics develops trunk muscular endurance [42]; however, this study indicates that young trained gymnasts can benefit from an alternative trunk endurance training stimulus. These results suggest that neuromuscular training which incorporates traditional gymnastics “shaping” exercises (e.g., dish/arch holds, handstand shaping), with isometric trunk endurance exercises (e.g., plank holds, flexor and extensor gravity holds) is more effective in eliciting improvements in trunk muscular endurance than sports-specific training alone.

Examination of the group mean data showed that the EXP group made non-significant improvements in leg stiffness, while the CON groups’ leg stiffness significantly decreased. Furthermore, neither group of gymnasts made significant improvements in RSI. These results indicate that on a group level, while the neuromuscular training failed to make significant improvements in performance, it was at least in part able to maintain leg stiffness and reactive strength qualities during the competitive period, where cumulative fatigue and detraining are more likely to occur. Previous research in elite pre-pubertal gymnasts showed gymnasts were unable to maintain gains in drop jump performance (from previous plyometrics and resistance training), confirming detraining following a competitive and transition period [5]. Therefore, it is possible that in the current study, accumulated fatigue blunted the training response for the gymnasts in the EXP group.

While the group results provide some insight into the effectiveness of the neuromuscular training intervention, they fail to differentiate between gymnasts that were positive responders, non-responders, and negative responders. In terms of the individual response to training for movement proficiency, a greater number of individuals in the training group responded positively in all three tests compared with the CON group, and the total number of positive responders was significantly higher in two (deep overhead squat and left in-line lunge [left leg lead]) of the three tests. Interestingly, 14/17 of the individuals in the EXP group were right-leg dominant, which could indicate that the neuromuscular training was effective in starting to address lower-limb asymmetry that could have developed due their sports-specific training [11,26]. These findings suggest that neuromuscular training targeting the development of simple fundamental movement skills can improve movement proficiency in pre-pubertal female gymnasts, beyond sports-specific training alone. This is somewhat unsurprising and consistent with previous research in pre-pubertal youth (13, 38), given that fundamental movement skills are particularly trainable in the pre-pubertal years [10,43] owing to the natural increases in neural plasticity that occur at this stage of development [44].

Examination of the individual responsiveness for the extensor muscular endurance test determined that the neuromuscular training intervention resulted in significantly more positive responders (76%), when compared to the CON (29%). Interestingly, the distribution of positive responders, non-responders and negative responders for the flexor muscular endurance in the EXP group (88%, 6%, 6% versus 53%, 18%, 29%) suggests that the EXP group responded more favourably than the CON. However, the number of positive responders in the EXP group was not significantly higher than in the CON. Similar results have previously been noted in mature female gymnasts, which investigated the effects of a 10-week trunk muscular endurance training program (two 15-min sessions per week) on reducing incidences of low-back pain. The study showed that the training group significantly improved lateral flexor, extensor and flexor endurance, while the CON group only demonstrated improvements in flexor endurance [33]. Cumulatively, the results indicate that gymnastics-training may favour the development of flexor muscular endurance; however, supplementary training programs might be necessary to target lateral and extensor muscular endurance. Furthermore, research shows that isometric training can enhance core/torso stiffness, which as a result, increases athletes’ load bearing capacity and improves force transfer during ballistic distal limb movement, albeit in adults [45,46]. These adaptations could be highly beneficial and relevant to young gymnasts’ from both a performance and injury risk perspective; however, further research is needed to explore these potential benefits in this population.

Individual responsiveness data revealed that significantly more gymnasts in the EXP group elicited positive adaptations in leg stiffness (41%), when compared to controls (12%). However, when examining the number of positive responders versus the number of trivial and negative responders in the EXP group, the results indicate that the training stimulus only promoted improvements in leg stiffness for approximately 50% of gymnasts. Individuals who demonstrated improvements in RSI were not significantly greater than the expected count in either group. Furthermore, the distribution of positive responders, non-responders and negative responders for RSI (EXP = 53%, 23.5%, 23.5% versus CON = 65%, 6%, 29%) suggests that the addition of the current neuromuscular training program did not provide a superior stimulus to the gymnastics training alone. The differentiated individual responsiveness among both EXP and CON groups highlights the unique aspects of the sport of gymnastics, whereby the inherent physical demands of training and competing are likely to promote a degree of adaptation in various indices of SSC function [47].

Previous research has shown that pre-pubertal gymnasts are superior in jumping tasks when compared to young athletes from other sports [48]. A training study designed to improve jumping performance in elite pre-pubertal gymnasts’, showed that two sessions per week (90 min each) of heavy resistance training and high impact plyometrics was effective in improving drop jump performance (from 20, 40, 60 cm heights) parameters (flight time, contact time, estimated mechanical power and flight: contact ratio) [5]. In the present study, the training program incorporated a number of hopping and skipping-based plyometric exercises, instead of more eccentrically demanding exercises such as drop jumps or maximal hopping. Therefore, it is suggested that the positive responders in the EXP cohort experienced specific adaptations to the imposed demands. For those that did not respond positively, it could be postulated that the training stimulus provided was not sufficiently intense to promote improvements in fast-SSC (e.g., reactive strength) in the pre-pubertal gymnasts. A recent meta-analysis concluded that the most effective dose-response relationship for young athletes occurred with conventional resistance training programs of periods >23 weeks, 5 sets per exercise, 6–8 repetitions per set, a training intensity of 80%–89% of 1 RM [49]. Thus, young gymnasts may require higher intensities and longer training durations to elicit improvements in more physically demanding movement tasks. Alternatively, gymnastics training itself might be a sufficient stimulus to improve reactive strength qualities in certain individuals; however, long-term exposure to neuromuscular training may increase gymnasts’ athletic development and reduce their sport-related injury risk [11].

Some limitations should be noted in this study. Firstly, participant characteristics between the two groups were significantly different at baseline, with the CON group slightly more mature by approximately 1 year. However, a MANCOVA revealed no significant differences between the groups’ performance variables at baseline. Whether this difference in biological maturity would have influenced the post-testing findings despite both groups being pre-PHV is unclear and warrants further investigation. Secondly, as the current study took place over a competitive phase for the gymnasts, it is possible that accumulated fatigue influenced the results by blunting the training response for the gymnasts in the EXP group. Future research should aim to monitor workloads using rate of perceived exertion scales in both CON and EXP groups that are specific to youth [50]. Furthermore, the current study only used an 8-week training intervention and primarily field-based measures of neuromuscular performance to evaluate the effectiveness of the training program. Owing to the nature of the young artistic gymnasts’ population, future research is warranted that examines the effects of longer-term exposure to strength and conditioning activities, using more advanced strength and power testing diagnostics. Notwithstanding these limitations, this study has important strengths. Despite the high injury rates in young artistic gymnastics [51] and the risk reducing benefits of strength and conditioning for young female athletes [33,52], there is a dearth of published literature examining individual training responsiveness of this population to neuromuscular training. This exploratory study offers a novel contribution to the literature by providing researchers and practitioners with insight into the differentiated responsiveness of young female gymnasts to a neuromuscular- and gymnastics-training stimulus.

## 5. Conclusions

The results from this study show that a neuromuscular training programme can provide additional benefits to some pre-PHV gymnasts, beyond gymnastics-training programs alone. With just 2 × 35-min sessions per week, strength and conditioning coaches can enhance trunk muscular endurance and movement proficiency in a relatively short period of time. For gymnasts to realise ongoing adaptations, exercises will need to be progressively overloaded and training prescription will need to be altered. Strength and conditioning practitioners should also be aware that providing technical competency is sufficiently robust, pre-PHV gymnasts may require higher exercise intensities (i.e., larger external resistance) and longer training durations to facilitate neuromuscular adaptations that improve reactive strength, leg stiffness and fast-SSC function. Importantly, strength and conditioning practitioners must prioritise technical competency at all times and aim to differentiate exercises on an individual basis via regressions and progressions.

## Figures and Tables

**Table 1:** Anthropometric measures for the training and control groups.

Group	Age (Year)	Body Mass (kg)	Standing Height (cm)	Seated Height (cm)
Training	8.2 ± 1.7	26.1 ± 5.1	127.0 ± 10.0	68.5 ± 5.3
Control	10.0 ± 1.2 *	30.8 ± 5.4 *	134.0 ± 9.2 *	70.4 ± 4.1

* Significantly greater than the training group (*p* < 0.05).

**Table 2:** Overview of the 8-week INT training programme.

W		Exercise	Sets	Reps
1	TC	Dish, arch & plank conditioning set	2	10
	MP	Inch worms, gluteal bridges, bilateral CMJs, broad jumps,		
	RT	Countermovement squats, split squats, forward lunges,		
2	TC	Dish, arch & support conditioning set	2	10
	MP	Clams, SL gluteal bridges, bilateral CMJs, broad jumps,		
	RT	Back squats *, split squats, forward lunges,		
3	TC	Extension, flexion & lateral isometric holds, plank isometric holds	4	6
	MP	Monster walks, SL gluteal bridges, rebounds, skipping, hop & stick		
	RT	Goblet squats, drop landing, SL lunges (DB), SL rock to stand		
4	TC	Extension, flexion & lateral isometric holds, plank isometric holds	4	6
	MP	Monster walks, SL gluteal bridges, rebounds, skipping, hop & stick		
	RT	Goblet squats, drop landing, SL lunges (DB), SL rock to stand		
5	TC	Extension, flexion & lateral isometric holds, plank isometric holds	4	6
	MP	Abductor leg lifts, Hamstring bridges, multidirectional hop & stick, pogos		
	RT	Goblet squats, drop landing, SL lunges (DB), SL rock to stand		
6	TC	Handstand trunk conditioning set	3	5
	MP	Abductor leg lifts, Hamstring bridges, multidirectional hop & stick, pogos		
	RT	Overhead squats *, RDL, SL squat to box		
7	TC	Handstand trunk conditioning set	3	5
	MP	Monster walks (MB), hamstring bridge feet raised, hop hop stick, pogos		
	RT	Overhead squats *, RDL (RT), SL squat to box		
8	TC	Dish, arch & support conditioning set (loaded)	3	5
	MP	Monster walks (MB), hamstring bridge feet raised, hop hop stick, pogos		
	RT	Overhead squats (RB), RDL (DB), SL squat to box		

W = week; TC = Trunk conditioning; MP = Movement preparation; RT = Resistance training; SL = single leg; CMJ = countermovement jumps; * = wooden dowel; MB = mini band; DB = dumbbell; RB = resistance band.

**Table 3:** Group mean and percentage changes in performance variables for both groups.

	Extensor End.				Flexor End.				Stiffness				RSI			
	Pre	Post	Group Δ%	*n* > SWC	Pre	Post	Group Δ%	*n* > SWC	Pre	Post	Group Δ%	*n* > SWC	Pre	Post	Group Δ%	*n* > SWC
Exp	72.0 ± 45.9	102.9 ± 44.6 *	28.5 ± 29.7 *	13 ^#^	100.5 ± 72.0	179.2 ± 95.8 *	38.7 ± 33.0 *	15	20.2 ± 4.6	20.8 ± 4.8	1.82 ± 15.69 *	7 ^#^	0.78 ± 0.30	0.94 ± 0.56	0.89 ± 48.01	9
Con	89.4 ± 25.6	92.5 ± 22.4	0.3 ± 26.5	5	119.1 ± 28.3	129.4 ± 58.33	−3.7 ± 47.7	9	23.9 ± 4.8	19.5 ± 4.8 ^	−27.63 ± 30.72	2	0.84 ± 0.38	0.95 ± 0.29	7.21 ± 40.86	11

Ext = Experimental group; Con = Control group; End. = endurance; RSI = reactive strength index; SWC = smallest worthwhile change; Δ = change in group; * = significantly greater than the Con (*p* < 0.05); ^ = significantly less than pre-testing (*p* < 0.05); ^#^ = significantly greater number of positive responders than the expected count compared to the control group.

**Table 4:** Group deep overhead squat and in-line lunge median results.

	Deep Squat					In-Line Lunge Left					In-Line Lunge Right				
Exp	Median pre		Median post		*n* > SWC	Median pre		Median post		*n* > SWC	Median pre		Median post		*n* > SWC
	1		2		13#	1		2		11#	1		2		8
	% of 1	52.9	1	0		% of 1	76.5	1	17.6		% of 1	64.7	1	29.4	
	% of 2	23.5	2	52.9		% of 2	5.9	2	58.8		% of 2	23.5	2	47.1	
	% of 3	23.5	3	47.1		% of 3	17.6	3	23.5		% of 3	11.8	3	23.5	
Con	Median pre		Median post		*n* > SWC	Median pre		Median post		*n* > SWC	Median pre		Median post		*n* > SWC
	2		2		5	2		2		3	2		2		6
	% of 1	35.3	1	11.8		% of 1	41.2	1	35.3		% of 1	29.4	1	5.9	
	% of 2	35.3	2	52.9		% of 2	41.2	2	35.3		% of 2	41.2	2	52.9	
	% of 3	29.4	3	35.3		% of 3	17.6	3	29.4		% of 3	29.4	3	41.2	

## Appendix A

Leg stiffness (kilonewtons per meter) was calculated using measures of body mass, contact times, and flight times. Within the equation, *K_N_* refers to leg stiffness, *M* is the total body mass, *T_c_* is equal to ground contact time, and *T_f_* represents the flight time [18,31].

(A1) $$K_{N}=\left[Μ\times \pi \left(T_{f}+T_{c}\right)\right]/T_{c2}\left[\left(T_{f}+T_{C}/p\right)-\left(T_{c}/4\right)\right]$$

RSI (millimeters per milliseconds) was calculated using jump height and contact time, with jump height calculated via flight time [35,36].

RSI (mm/ms) = jump height (millimeters)/contact time (milliseconds)
(A2)
